# A comprehensive whole genome database of ethnic minority populations

**DOI:** 10.1038/s41598-024-63892-1

**Published:** 2024-06-17

**Authors:** Yan He, Changgui Lei, Chanjuan Wan, Shuang Zeng, Ting Zhang, Fei Luo, Ruichao Li, Xiaokun Li, Anshu Zhao, Defu Xiao, Yunyan Luo, Keren Shan, Xiaolan Qi, Xin Jin

**Affiliations:** 1 Key Laboratory of Endemic and Ethnic Diseases, Ministry of Education and Key Laboratory of Medical Molecular Biology of Guizhou Province, Collaborative Innovation Center for Prevention and Control of Endemic and Ethnic Regional Diseases Co-constructed by the Province and Ministry, Guizhou Medical University, Guiyang, 550004 Guizhou China; 2 BGI Research, Shenzhen, 518083 China; 3 Present Address: BGI Research, Guiyang, 550000 China; 4 BGI Research, Wuhan, 430074 China; 5 Shenzhen Key Laboratory of Transomics Biotechnologies, BGI Research, Shenzhen, China; 6 School of Medicine, South China University of Technology, Guangzhou, China

**Keywords:** GMGD database, Human whole genome sequencing, Guizhou Province in southwest China, Ethnic minority populations, Computational biology and bioinformatics, Genetics

## Abstract

China, is characterized by its remarkable ethnical diversity, which necessitates whole genome variation data from multiple populations as crucial tools for advancing population genetics and precision medical research. However, there has been a scarcity of research concentrating on the whole genome of ethnic minority groups. To fill this gap, we developed the Guizhou Multi-ethnic Genome Database (GMGD). It comprises whole genome sequencing data from 476 healthy unrelated individuals spanning 11 ethnic minorities groups in Guizhou Province, Southwest China, including Bouyei, Dong, Miao, Yi, Bai, Gelo, Zhuang, Tujia, Yao, Hui, and Sui. The GMGD database comprises more than 16.33 million variants in GRCh38 and 16.20 million variants in GRCh37. Among these, approximately 11.9% (1,956,322) of the variants in GRCh38 and 18.5% (3,009,431) of the variants in GRCh37 are entirely new and do not exist in the dbSNP database. These novel variants shed light on the genetic diversity landscape across these populations, providing valuable insights with an average coverage of 5.5 ×. This makes GMGD the largest genome-wide database encompassing the most diverse ethnic groups to date. The GMGD interactive interface facilitates researchers with multi-dimensional mutation search methods and displays population frequency differences among global populations. Furthermore, GMGD is equipped with a genotype-imputation function, enabling enhanced capabilities for low-depth genomic research or targeted region capture studies. GMGD offers unique insights into the genomic variation landscape of different ethnic groups, which are freely accessible at https://db.cngb.org/pop/gmgd/.

## Introduction

The advances in DNA sequencing technology and decreasing costs have allowed for unprecedented exploration of human genetic diversity. The analysis of genome sequencing data from diverse human populations can identify novel human variants, establish genotype frequency baselines, and elucidate population genetic structures and evolution. These investigations can provide precise and comprehensive insights into the genetic landscape of various ethnicities, laying a crucial foundation for our understanding of population differences, admixture, and guiding the development of new drugs^1,2^. Despite the potential of these advances, the representation of certain populations and ethnic groups, particularly those in Southwest China, remains minimal in large-scale genetic databases.

Southwest China, housing 31 distinct ethnic minorities^3^, encompassing the provinces of Sichuan, Chongqing, Guizhou, Yunnan, and Tibet, it is characterized by its rich genetic diversity and complex genetic history. Due to the region’s unique geographical location and historical factors, these groups have been able to preserve their distinct and representative genetic characteristics^4^. Previous genotyping studies on Y chromosome short tandem repeat loci (STR)^5^, autosomal insertion/deletion polymorphisms (InDels)^6^, and whole genome single nucleotide polymorphisms (SNPs) in ethnic minority populations^7^ have underscored the significant research value of genetic information of these groups.

However, in spite of this, the representation of Southwest China’s population in large-scale gene databases remains minimal, with even scarcer information on specific ethnic groups^8–10^. For instance, the recently published large population cohort, like NyuWa genomes and ChinaMap, includes less than 10% of the Southwest population without ethnic information. Moreover, the Human Genome Diversity Project (HGDP) database, as important reference data in population genetics research, provides a limited representation with only 10 samples from three ethnic groups: Miao, Yi, and Tujia.

These gaps underline a significant lack of representation and lack of ethnic-specific data, which hinders our full understanding of our species' genetic diversity. Therefore, there is an urgent need to establish a comprehensive genetic resource encompassing these underrepresented minority populations. By doing so, we not only facilitate data exchange and resource sharing but also advance frontier exploration in research and foster industrial transformations.

In this study, we performed whole genome sequencing (WGS) of 476 male individuals representing 11 ethnic minorities from Southwest China. Utilizing the genome analysis data, we constructed the Guizhou Multi-ethnic Genome Database (GMGD), aiming to fill the current gap in genetic data representation. This database offers a search method for the genomic region or point mutations and displays the frequency differences of mutations across various populations.

To further enhance the utility of GMGD, we employed Principal Component Analysis (PCA) and admixture analyses to illustrate population structure among ethnic groups. Furthermore, we provided a genotype-imputation function, which could serve as a valuable tool for other genomic studies. Thus, GMGD, with these features, constitutes a significant step toward understanding of the evolutionary and medical implications of different ethnic groups in Southwest China.

## Materials and methods

### Sample collection

GMGD database was constructed using the results of whole genome data derived from 476 unrelated, healthy male individuals belonging to various ethnic minorities from Guizhou Province, southwest China. From 1999 to 2002, the project team successively visited 10 counties and 24 townships in Qiandongnan Miao and Dong Autonomous Prefecture, Qiannan Buyi and Miao Autonomous Prefecture, Tongren City and Bijie City of Guizhou Province, conducted investigation and research on the local ethnic minorities, and collected thousands of DNA samples based on informed consent. A DNA sample bank of Guizhou ethnic minorities was established. In this study, suitable samples were selected from the sample bank. In our selection criteria, all individuals who lived in Guizhou, were ethnically homogeneous within three generations, and did not marry outside the population, had no blood with each other The relationship. The participants represented 11 ethnic minorities from three language branches: Hmong–Mien languages (Miao, n = 94; Yao, n = 30)), Tai-Kadai languages (Dong, n = 40; Bouyei, n = 53; Gelao, n = 39; Zhuang, n = 30; Sui, n = 40), and Sino-Tibetan languages (Yi, n = 40; Bai, n = 30; Tujia, n = 39); Hui, n = 40) (Table 1).

**Table 1:** The information on samples collection.

Language branch	Ethnic	No. of samples	Location
Hmong–Mien languages	Miao	94	Bijie; Qiannan;Qiandongnan
	Yao	30	Qiannan
Tai–Kadai languages	Dong	40	Qiandongnan
	Bouyei	53	Bijie; Qiannan
	Gelao	39	Bijie
	Zhuang	30	Qiandongnan
	Sui	40	Qiannan
Sino-Tibetan languages	Yi	40	Bijie
	Bai	30	Bijie
	Tujia	39	Tongren
	Hui	40	Bijie

This study was undertaken according to the Measures for the Ethical Review of Life Science and Medical Research Involving Humans. This study was supported by the Research Ethics Committee of the Affiliated Hospital of Guizhou Medical University (IRB No. 2021201), and before participating in this study, all individuals were required to complete an informed consent form. An informed permission form was signed by each of the recruited volunteers.

### Sequencing library construction and reads alignment

Upon receiving signed informed consent from the donors, we collected blood samples from each participant. Genomic DNA was extracted from these samples and sequenced on the DNBSEQ-T1 platform. Following are the steps taken to generate a whole-genome library: (1) Covaris was used to randomly fragment DNA, and then magnetic beads were used to screen genomic DNA fragments with an average size of 200–400 bp. (2) Terminal repair of DNA fragments was performed, followed by 3′ adenylation. Adaptors were attached to the ends of these 3′ adenylated fragments by T4 DNA ligase. (3) PCR was used to amplify genome products. (4) A qualified genome library was constructed and sample quality checked prior to sequencing with paired-end 100 bp reads on the DNBSEQ T1 platform with an average depth of 5.5 ×. To evaluate the quality of genotype calling, we increased the sequencing depth to 30 × for a subset of 34 individuals. For each sample, the cleaned reads were aligned to the GRCh37 and GRCh38 human genome reference using BWA, with the paired-end read alignment option^11^. Following the initial data quality control, the samtools rmdup module to remove duplicates, and GATK (v3.8) to recalibrate the base quality score^12,13^ We have identified duplicate samples using the—genome function of Plink version 1.9, and we have calculated the sequencing depth, GC content, Q20, and Q30 quality metrics for each sample, with the relevant details provided below. See Supplementary Fig. S1 for further information.

### Variant calling and filtering

After removing duplicates and recalibrating the base quality scores (BQSR), variants were called using the Haplotyper algorithm in GVCF mode. Subsequently, the GVCFtyper algorithm was utilized to perform joint-calling and generate the cohort VCF. Variant Quality Score Recalibration (VQSR) was conducted using the Genome Analysis Toolkit (GATK version 4.1.2). The truth-sensitivity-filter-level was set to 99.1 for SNPs and 99.8 for Indels.

To ensure high quality, we applied several filters to exclude low-quality variants. First, variants that did not pass VQSR were removed. Then, we eliminated sites with genotype quality (GQ) < 10 in more than 50% of samples. Additionally, variants showing excessive heterozygosity or with ExcessHet > 54.69 in the INFO column, calculated by GATK, were filtered out. We also removed variants that were closer to an INDEL, using the bcftools filter command with the options—SnpGap 3 and—IndelGap 5.

### Assessment of genotype calling accuracy

We utilized sequencing and genotype data from 34 individuals (30 ×) to assess the accuracy of GMGD low-depth WGS genotype calling. The genotype obtained from high coverage WGS was considered as the true genotype, while the called variants served as the test set. Variants with calling rates exceeding 95% per sample and a frequency above 1% were preserved. Ultimately, we estimated genotype concordance by considering only the variants in autosomes that were identified by both low WGS and high WGS, encompassing NR Sensitivity and NR genotype concordance. In addition to this, we also downsampled 50 30X high-coverage samples from the 1000 Genomes Project to 1 ×, 5 ×, and 15 × to conduct corresponding data validation and calculate genotype concordance.

### Variant annotation and population structure analysis

The high-confidence variants were used for downstream analyses and statistics. We utilized the Ensembl Variant Effect Predictor (VEP) in conjunction with the relevant VEP-compiled annotation database to annotate the variants^14^. To display the genetic substructure between different ethnic branches, PCA and admixture were constructed by using PLINK (v1.9) and ADMIXTURE analyses^15,16^.

### Reference panel construction and Evaluation for imputation

We employed BEAGLE version 5.2 to conduct haplotype phasing of all 476 samples, utilizing the default settings. The chromosome was partitioned into overlapping chunks of 1 Mb in size, with a 0.1 Mb overlap between adjacent chunks. Then we evaluated the accuracy of genotype imputation for GMGD panels by using 50 Chinese populations in 1 KG.

### Database implementation

GMGD is available at https://db.cngb.org/pop/gmgd/. The specific IT strategies are as follows: Considering the open source and popularity, flask (https://flask.palletsprojects.com/) and MongoDB (https://www.mongodb.com/) were chosen as the backend framework and the database engine, separately. The VUE framework (https://vuejs.org/) was used to develop the web frontend interface. To achieve web functions, we utilized JQuery, a versatile and comprehensive JavaScript library, along with Bootstrap, an open-source toolkit for web development, as plugins for our project. Furthermore, our website incorporated D3, a JavaScript library for data-driven document manipulation, and Echart, an open-source toolkit for data visualization, to effectively visualize genomics data.

### Ethics approval and consent to participate

This study design and procedures were approved by the Institutional Review Board (IRB) of Guizhou Medical University of China (IRB No. 2021201), and before participating in this study, all individuals were required to complete an informed consent form. An informed consent form was signed by each of the recruited volunteers.

## Results

### Variants identification

By aligning to different reference genomes, a total of 16,336,982 variants (Ts/Tv ratio = 2.11) were filtered in GRCh38, of which 11.9% (1,956,322) are entirely new and do not exist in the dbSNP database. Similarly, a total of 16,205,829 variants (Ts/Tv ratio = 2.12) were filtered in GRCh37, with 18.5% (3,009,431) of these variants being entirely new and not present in the dbSNP database.

**Figure 1:**
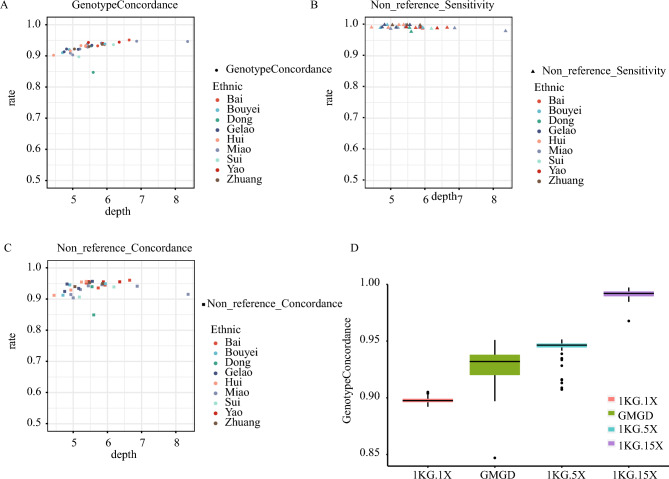
Genotype Evaluation of the GMGD WGS data. (**A**) Genotype concordance rate versus sequencing depth for 33 GMGD samples in different Ethnic. (**B**) Non reference Sensitivity rate versus sequencing depth for 33 GMGD samples in different Ethnic. (**C**) Non reference Concordance rate versus sequencing depth for 33 GMGD samples in different Ethnic. (**D**) Genotype concordance rates for 34 GMGD samples and downsampling of 50 samples from the 1000 Genomes Project.

Assuming the high-depth WGS data as the gold standard, we achieved that the genotype concordance and non-reference (NR) genotype concordance rate extended to 0.951 and 0.9606 with increasing sequencing depth (Fig. 1A,B). The NR sensitivity even reaches 0.9907 (Fig. 1C). Additionally, downsampling was performed on 50 30X high-coverage samples from the1000 Genomes Project, and the results showed that genotype concordance is positively correlated with sequencing depth. Moreover, the genotype concordance rate is quite similar when the sequencing depths are comparable (Fig. 1D). These statistical data indicate the high quality of our mutated dataset.

### Search for variants (genomes)

**Figure 2:**
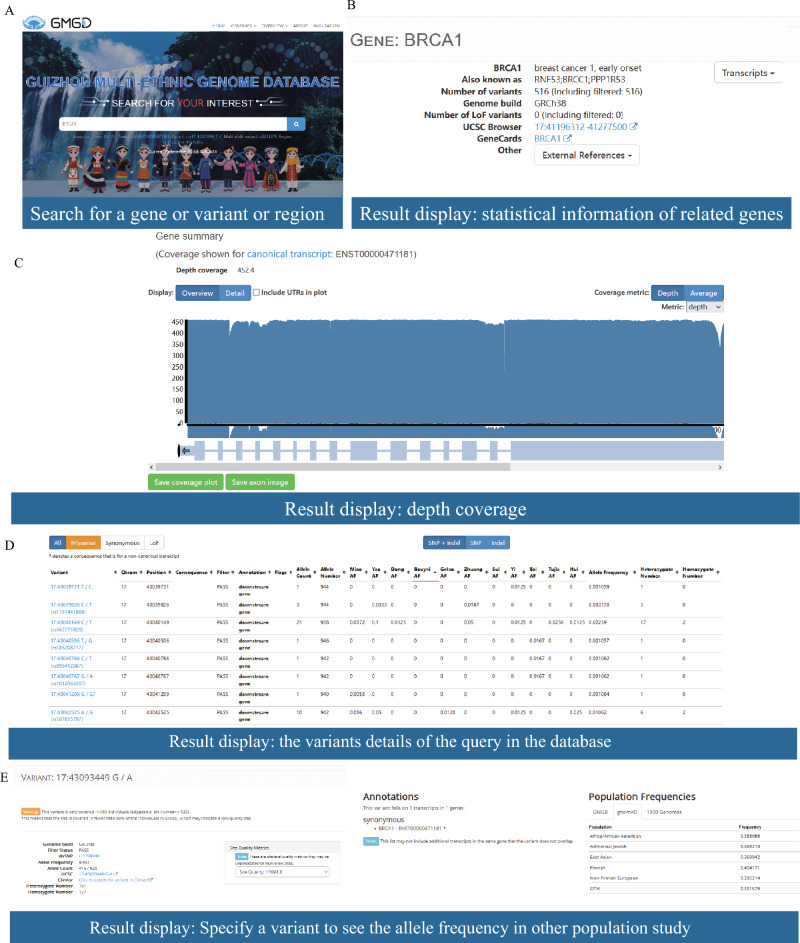
Screenshots of the BRCA1 gene search and its corresponding search results are shown below. (**A**) The BRCA1 gene was used as an example for the search, where the corresponding transcript, variant, and region can all be used to obtain a similar results page. The following shows the query results. (**B**) The gene summary includes the number of variants and a UCSC browser link. (**C**) A coverage plot for a transcript is displayed. (**D**) Details of variants queried in the database are shown. (**E**) It is possible to specify a variant to view the allele frequency in other population studies.

GMGD provides users with five methods to search for variant information based on two reference genome versions of GRCh37 and GRCh38: Gene, Transcript, Variant, Multi-allelic variant, and genomic Region that spans not more than 100 kb (Fig. 2A).

For example, researcher can search for *BRCA1*, one of the most common breast cancer genes, on the home page and get the gene summary (Fig. 2B) including the coverage plot of the query transcripts or the genomic regions (Fig. 2C) and corresponding variants data comprising of chromosome positions, annotation, allele frequency (Fig. 2D), etc. We also provided relevant information on all variations including population frequency among different ethnic groups to discover ethnic-specific variations quickly (Fig. 2E). Search in any method, all the relevant elements will be displayed for understanding of the corresponding relationships between different elements and achieving interactive retrieval. users can click on the variations they pay close attention to enter a more detailed information page.

### Overview page

**Figure 3:**
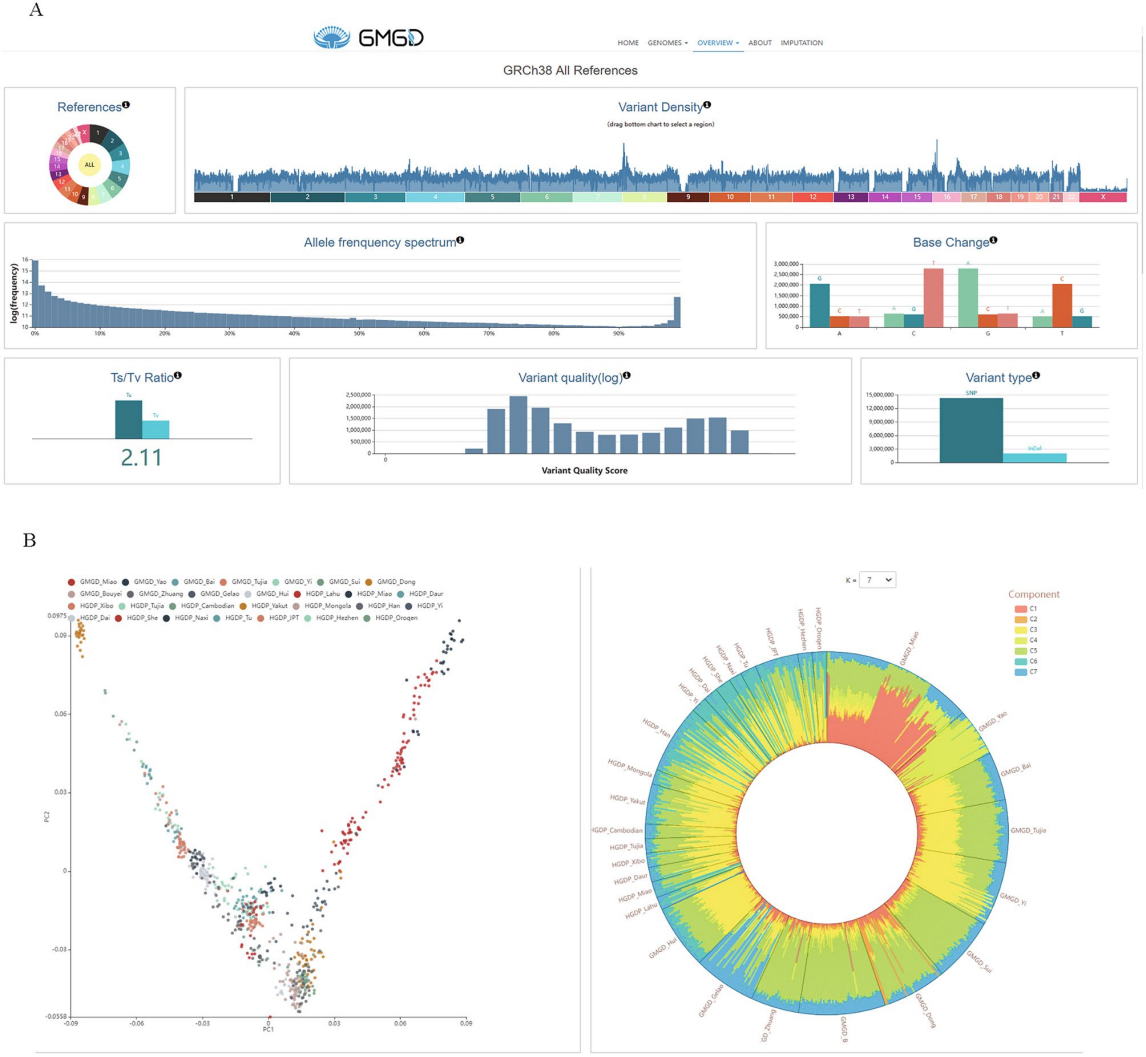
Overview page detail of the GMGD Database. (**A**) Gnome statistics information, which includes variant density, allele frequency spectrum, base change, Ts/Tv ratio, variant quality, and variant type in GRCh38 reference genome versions. (**B**) PCA and admixture displayed on the overview page.

The overview page displays genome statistics information, which includes variant density, allele frequency spectrum, base change, Ts/Tv ratio, variant quality, and variant type. The distribution of variation on all chromosomes is calculated based on two reference genome versions: GRCh37 and GRCh38. The user-friendly interfaces provide selection functionality for users to select reference genome versions by clicking on the dropdown box or chromosome regions of interest by clicking the color block of references or the horizontal axis of the variant density (Fig. 3A).

Principal Component Analysis (PCA) and Admixture analyses displayed on the overview page show that the clustering model of the Guizhou population is consistent with the linguistic classification of populations from different regions around the world. Interactive features assist users in understanding the structural relationships of various ethnic groups on a global scale. Clicking on a population in the PCA legend allows for hiding or accessing each population. Zooming in on a specific area can be done by using the scroll function on the mouse. Different k-values can be selected from the dropdown menu above the Admixture analysis. Additionally, the proportion of each ancestral component will be displayed when the mouse hovers over a sample (Fig. 3B).

### Imputation development

**Figure 4:**
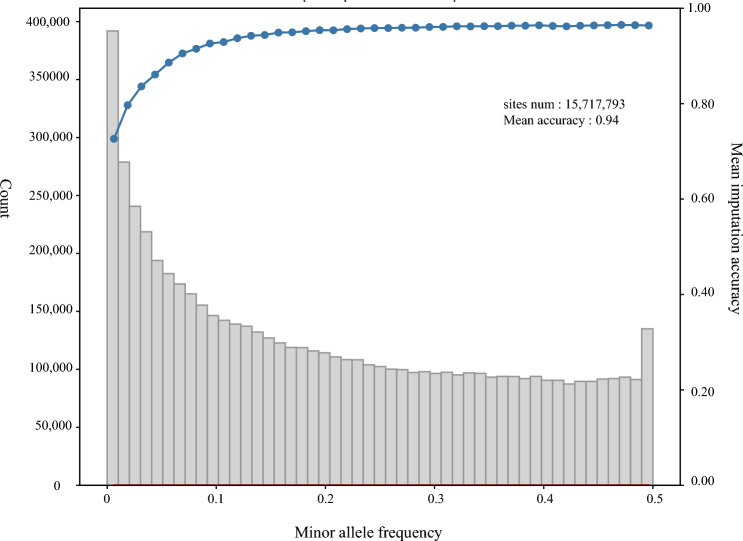
The histogram of imputed variants and Pearson correlation coefficients for GMGD panel.

In evaluating the imputation quality of the GMGD panel, we conducted a comparison of the squared correlation coefficient (R^2^) between the genotypes identified in the high coverage data of the 50 Chinese Southern Han in the 1000 Genomes Project and the imputed genotypes in the array data. We filtered for variants with an info score > 0.4 and a *p* value from a chi-square test of Hardy–Weinberg equilibrium larger than 10^–6^. Ultimately, a total of 15,717,793 variants were effectively imputed. The mean imputation accuracy for these variants was 0.94, indicating the high quality of the GMGD panel imputation (Fig. 4).

To further facilitate genotype imputation among the Chinese population and ethnic minorities, we developed a user-friendly imputation server (https://db.cngb.org/imputation/) for public use. This platform allows users to freely register, create imputation jobs, and upload their VCF-formatted array data in a bgzipped format, all within a secure data environment.

## Discussion

Establishing a comprehensive multi-ethnic genome resource platform will help to understand the diversity of human genetic resources around the world. The large-scale genetic data such as The 1000 Genomes Project ^17^, China Metabolic Profiling Project (ChinaMAP)^8^, NyuWa genome^18^, Westlake BioBank for Chinese (WBBC)^9^ pilot project and Taiwan Biobank^19^ have remodeled fine-scale genetic profiles of the major populations in China as well as a detailed framework of the population evolutionary history, which provided new opportunities for Chinese genetic research in health and disease, there are relatively few samples in the entire southwestern region. The genome reference sequences of ethnic minorities are extremely scarce.

GMGD is currently the genome database for the largest number of ethnic groups. Aiming to fill the current gap in genetic data representation. Compared with commonly used reference genome database such as HGDP, GMGD have more genome samples and abundant population diversity. For example, there are 10 Miao ethnic groups in HGDP and 94 in GMGD^20^. This is also the first complete genome resource of the Gelao ethnic group^21^. GMGD will greatly enrich the abundance and breadth of population genetic resources. Besides, we employed Principal Component Analysis (PCA) and admixture analysis to illustrate population structure among ethnic groups. and we developed an imputation server with a user-friendly website interface for public use (URL) it can To Efficient, Accurate facilitate genotype imputation for different ethnic populations will contribute to a more comprehensive and accurate understanding of human genetic diversity and population evolution history and research on related diseases. GMGD with these features, constitutes a significant step toward understanding of the evolutionary and medical implications of different ethnic groups in Southwest China. By doing so, we not only facilitate data exchange and resource sharing but also advance frontier explorations in research and foster industrial transformations.

Currently, our database includes 476 males, covers 11 ethnic groups, and provides genome-wide variation information. In the future, we plan to improve the database in the following areas. Firstly, we will expand the sample size to enroll more samples and ethnic groups to form a more comprehensive genetic resource of Chinese. Secondly, we will enrich the database content, such as CNV information, Y chromosome haplotype information, etc., to achieve a broader range of user interaction functions. Furthermore, integration and interaction with other databases will also become the direction of efforts.

In general, as the central repository of ethnic minority genomic data in China, GMGD will become a useful database and effective platform for the evolution of population genetics and medical research practices.

## Conclusion

The GMGD interactive interface facilitates researchers with multi-dimensional mutation search methods and displays population frequency differences among global populations. Furthermore, GMGD is equipped with a genotype-imputation function, enabling enhanced capabilities for low-depth genomic research or targeted region capture studies. GMGD offers unique insights into the genomic variation landscape of different ethnic groups, which are freely accessible at https://db.cngb.org/pop/gmgd/.

## Data availability.

Project name: GUIZHOU MULTI-ETHNIC GENOME DATABASE. Project home page: https://db.cngb.org/pop/gmgd/. The variation data reported in this paper have been deposited in the Genome Variation Map (GVM)^22^ in National Genomics Data Center, Beijing Institute of Genomics, Chinese Academy of Sciences and China National Center for Bioinformation^23^, under accession number GVM000478: https://ngdc.cncb.ac.cn/gvm.

### Supplementary Information

Supplementary Figure S1.
