# Author Correction: A comprehensive whole genome database of ethnic minority populations

**DOI:** 10.1038/s41598-024-66738-y

**Published:** 2024-07-09

**Authors:** Yan He, Changgui Lei, Chanjuan Wan, Shuang Zeng, Ting Zhang, Fei Luo, Ruichao Li, Xiaokun Li, Anshu Zhao, Defu Xiao, Yunyan Luo, Keren Shan, Xiaolan Qi, Xin Jin

**Affiliations:** 1 Key Laboratory of Endemic and Ethnic Diseases, Ministry of Education and Key Laboratory of Medical Molecular Biology of Guizhou Province, Collaborative Innovation Center for Prevention and Control of Endemic and Ethnic Regional Diseases Co‑constructed by the Province and Ministry, Guizhou Medical University, Guiyang, 550004 Guizhou China; 2 BGI Research, Shenzhen, 518083 China; 3 Present Address: BGI Research, Guiyang, 550000 China; 4 BGI Research, Wuhan, 430074 China; 5 Shenzhen Key Laboratory of Transomics Biotechnologies, BGI Research, Shenzhen, China; 6 School of Medicine, South China University of Technology, Guangzhou, China

Correction to: * Scientific Reports* https://doi.org/10.1038/s41598-024-63892-1, published online 17 June 2024.

The original version of this Article contained errors in the Funding section.

“Funding was provided by the Natural Science Foundation of China (Grant Nos. 32360154, 31560306), the Guizhou Science and Technology Support Project, Social Development Field in the form of a Grant [(2019) 2807]. And the project of Key Laboratory of Endemic and Ethnic Diseases, Ministry of Education, Guizhou Medical University (No. FZSW-2022-001). This study was supported by National Natural Science Foundation of China (No.32171441), National Key Research and Development Program of China (No.2023YFC2605400, No.2022YFC2502402), Guangzhou Basic and Applied Basic Research Foundation (No.202201010189), Open Project of State Key Laboratory of Respiratory Disease (No.SKLRD-OP-202309), the Innovation Platform for Academicians of Hainan Province (No.YSPTZX202118), Key-Area Research and Development Program of Guangdong Province (No.2023B0303040001), the Shenzhen Key Laboratory of Genomics (No.CXB200903110066A), Guangdong Provincial Key Laboratory of Human Disease Genomics (No.2020B1212070028), Guangdong Provincial Key Laboratory of Genome Read and Write (No.2017B030301011), and the China National GeneBank.”

now reads:

“Funding was provided by the Natural Science Foundation of China (Grant Nos. 32360154, 31560306, 82360813), the Guizhou Science and Technology Support Project (Social Development Field [2019] 2807), the Science and Technology Fund of Guizhou Provincial Health Commission (gzwkj2024-056), the project of Key Laboratory of Endemic and Ethnic Diseases, Ministry of Education, Guizhou Medical University (No.FZSW-2022-001), and Guizhou Provincial Science and Technology Program Project Grant (QKH Platform Talents-GCC[2023]035, QKH Platform Talents-CXTD[2023]003, QKH-ZK-2022-401). Guangzhou Basic and Applied Basic Research Foundation (No.202201010189), Open Project of State Key Laboratory of Respiratory Disease (No.SKLRD-OP-202309), the Innovation Platform for Academicians of Hainan Province (No.YSPTZX202118), Key-Area Research and Development Program of Guangdong Province (No.2023B0303040001), the Shenzhen Key Laboratory of Genomics (No.CXB200903110066A), Guangdong Provincial Key Laboratory of Human Disease Genomics (No.2020B1212070028), Guangdong Provincial Key Laboratory of Genome Read and Write (No.2017B030301011), and the China National GeneBank.”

The original Article has been corrected.

