# Supplementary material for: A comprehensive whole genome database of ethnic minority populations
Source: Sci Rep. 2024 Jun 17;14:13954. doi: 10.1038/s41598-024-63892-1 (PMC11183174; doi:10.1038/s41598-024-63892-1)
Supplement: Supplementary file 1 — Supplementary Figure S1. [file 41598_2024_63892_MOESM1_ESM.pdf]

Figure S1

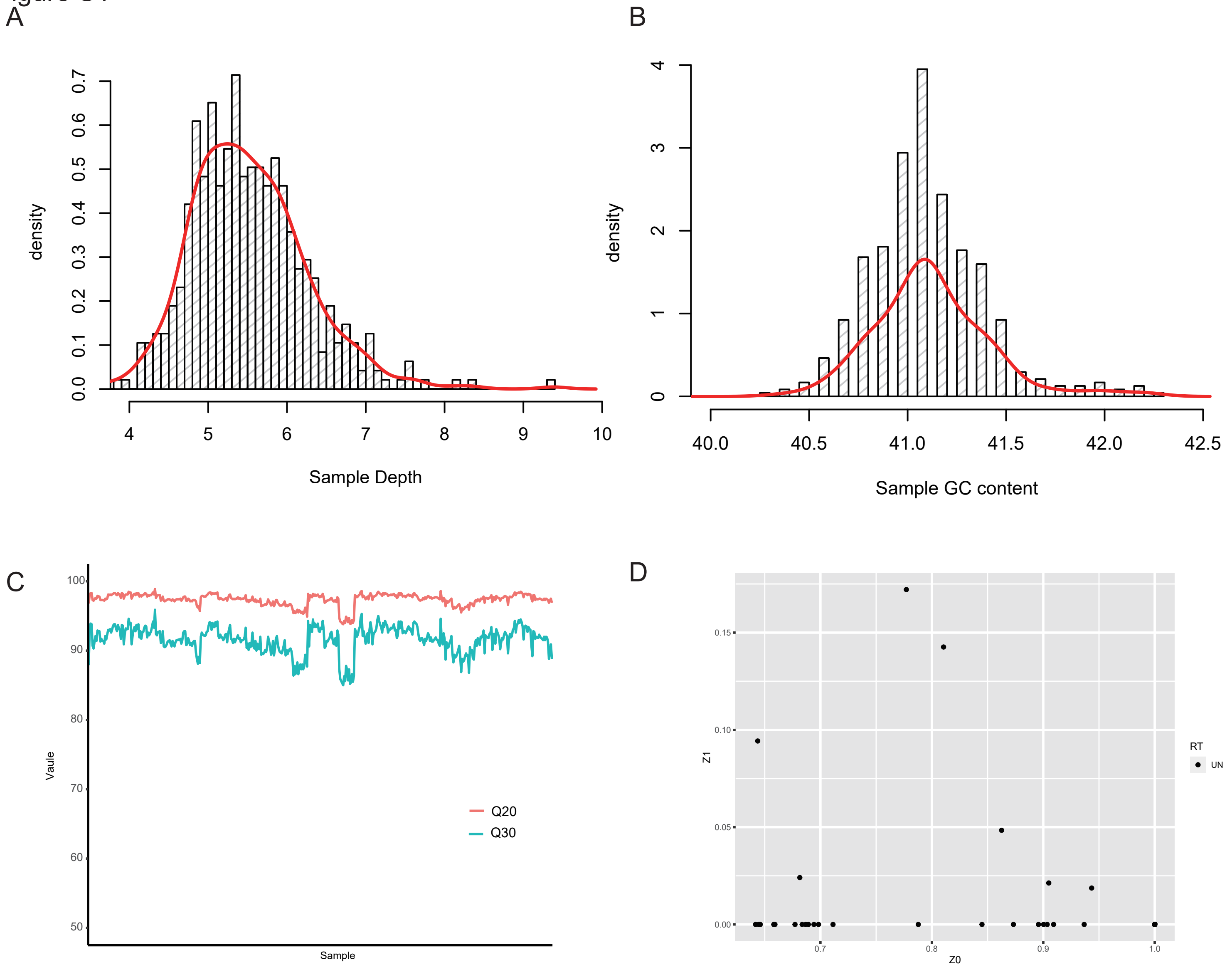

Supplementary Figure S1|Overview of Sequencing Characteristics and Quality Metrics for the Sampled Individuals.
(A) The sequencing depth for each sample
(B) The GC content for each sample
(C) The Q20, and Q30 quality metrics for each sample.
(D) IBD analysis verifying the non-relatedness of sampled individuals.
